# Molecular Link in Flavonoid and Amino Acid Biosynthesis Contributes to the Flavor of Changqing Tea in Different Seasons

**DOI:** 10.3390/foods11152289

**Published:** 2022-07-31

**Authors:** Qingping Ma, Mengyao Qin, Laichao Song, Haiwei Sun, Hong Zhang, Huanhuan Wu, Zhihong Ren, Hui Liu, Gang Duan, Yu Wang, Zhaotang Ding

**Affiliations:** 1 College of Agronomy, Liaocheng University, Liaocheng 252000, China; maqingping@lcu.edu.cn (Q.M.); qinmengyao202207@163.com (M.Q.); slc130564@163.com (L.S.); 2 Taian Academy of Agricultural Sciences, Taian 271000, China; shwhenry@163.com (H.S.); tazhanghong@163.com (H.Z.); hhwu0624@163.com (H.W.); rzhnky@163.com (Z.R.); 3 Jinan Bureau of Agriculture and Rural Areas, Jinan 271100, China; mytncp@163.com (H.L.); duang07@163.com (G.D.); 4 College of Horticulture, Qingdao Agricultural University, Qingdao 266109, China; wangyutea@163.com; 5 Tea Research Institute, Shandong Academy of Agricultural Sciences, Rizhao 276800, China

**Keywords:** Changqing tea, catechins, amino acids, flavonoid biosynthesis, metabolism

## Abstract

The present study was aimed to elucidate the flavor formation mechanism of Changqing tea. High-performance liquid chromatography (HPLC) analysis showed that the total catechins of Changqing tea was 65–160 mg/g, with 16–34 mg/g non-galloyated catechins and 49–126 mg/g galloylated catechins. Tea polyphenols and free amino acids account for 286–312 mg/g and 35–89 mg/g, respectively. Transcriptome of Changqing tea during different seasons revealed 316, 130 and 12 DEGs in comparisons of spring vs. autumn, spring vs. summer, and summer vs. autumn, respectively. Compared to spring, the genes involved in flavonoid biosynthesis and bitter imparted amino acids were up-regulated in summer and autumn. Metabolome analysis was conducted by using HPLC-MS; the result indicated that umami and kokumi contributing amino acids were decreased in summer and autumn compared with spring. It could be concluded that the coordination of flavonoid biosynthesis and amino acids biosynthesis resulted in the special flavor of Changqing tea.

## 1. Introduction

Tea plants (*Camellia sinensis*) are an important economic crop in China; their tender leaves are the source of the most popular tea drinks. It is well known that tea flavor could be affected by environment [1]. There are many famous high-quality green teas with different flavors originating in different places, such as Xihu Longjing (Hangzhou), Taiping Houkui (Huangshan), Huangshan Maofeng (Huangshan), and Dongting Biluochun (Suzhou) [2,3,4]. The excellent flavor of these famous green teas is caused by the different climates in the originating places.

Shandong Province belongs to the tea area north of the Changjiang River. There are thousands of years of history about tea cultivation from the Song Dynasty, and such teas were widely cultivated from as recently as 70 years ago. Now, there are many famous green teas produced in Shandong Province, such as Rizhao green tea, Laoshan green tea, and Yimeng green tea, etc. [5,6]. These teas are listed in geographical areas and are indicated as protected products now, and their growth areas are located in the eastern coastal region of Shandong Province, where it is warmer and wetter than other places in Shandong. With the progress of tea cultivation technology, tea plants have been planted in most regions of Shandong Province. Changqing is a district of Jinan in Shandong Province, located in the foot of Mount Tai. It has 60 years’ tea-planting history and is the highest latitude tea-growing area in China. Because of its unique geographical location, the air temperature is largely different between day and night (about 10 °C), as well as in different seasons. In addition, the water is of high quality with some beneficial minerals and low CaCO_3_ content, which is good for tea plant growth and tea flavor formation. It is the optimal organic tea cultivation and production area.

Now, Changqing tea is famous for its good quality, with thick, tender leaves, shiny and green dry tea and the chestnut flavor of the tea soup. However, the mechanism of Changqing tea formation remains unclear. The relationship between tea quality and the environment of Changqing tea needs to be elucidated. 

In this study, the transcriptome, metabolome, and flavor components of Changqing tea in different seasons were detected to explain its flavor formation mechanism. This study will also provide insights for understanding the tea quality formation mechanism of the tea area in northern China.

## 2. Materials and Methods

### 2.1. Plant Materials 

This study was conducted in two tea gardens, Nanhuyulu and Litaishan, in Changqing District in Jinan, the capital of Shandong Province (116°48′ E, 36°30′ N, 278 m above sea level). Both tea gardens are brown loam with a pH of 6.5, and are rich in minerals such as iron and copper. Tea shoots with one bud and two tender leaves from five-year-old tea plants in spring (4–6 May, 24 ± 4 °C day/13 ± 3 °C night), summer (7–9 July, 32 ± 3 °C day /23 ± 1 °C night), and autumn (23–25 September, 25 ± 1 °C day/16 ± 1 °C night) were harvested and frozen in liquid nitrogen for extraction of RNA and metabolites. All the samples were harvested at 9:00–10:00 a.m. in three consecutive sunny days, which were considered as three biological repeats.

### 2.2. Detection of Flavor Components of Changqing Tea

Total polyphenols (TP) were determined by TP detection kit (Yuanye, Shanghai, China) using the folinol colorimetry method, according to the manual. Free amino acids were detected by using ninhydrin colorimetry and L-Theanine was used as the standard. Catechins were detected by using the HPLC method following the instructions of GB/T8313-2018.

### 2.3. RNA Extraction and Illumina Sequencing

Total RNA was extracted by using an EASYspin Plus Plant RNA Extraction Kit (Aidlab Biotech, Beijing, China). The integrity, purity, and concentration of the RNA samples were detected by using a NanoDrop ND-1000 spectrophotometer (NanoDrop, Waltham, MA, USA) and 1% agarose gels. A total of 1 μg RNA was used for the cDNA library construction. The cDNA library construction and illumina sequencing were performed by BioMarker Biotech Ltd. (Beijing, China) according to protocols by Ma et al. [7].

### 2.4. Genome Alignment and Gene Prediction

After quality assessment, the clean reads of all the samples were mapped to chromosome-level genome of the tea plant [8] using HISAT2 software (http://ccb.jhu.edu/software/hisat2/index.shtml, accessed on 5 August 2020). The mapped reads were assembled and quantified by using String Tie (https://ccb.jhu.edu/software/stringtie/index.shtml, accessed on 5 August 2020). The unmapped transcripts were considered as new genes and annotated by NR [9], Swiss-Prot [10], GO [11], COG [12], KOG [13], Pfam [14], and KEGG databases [15] using BLAST [16]. 

### 2.5. Differential Expression Analysis

Pearson’s correlation coefficient was used to assess the variability of biological replicates. R^2^ close to 1 represents high correlation between biological replicates. Fragments Per Kilobase of transcript per Million fragments mapped (FPKM) was used to assess the expression level of genes. Differential expression analysis was conducted using DESeq2 [17], and fold change ≥1.5 and false discovery rate < 0.01 was used as the threshold. The classification of differential expressed genes (DEGs) were performed based on GO and KEGG databases. 

### 2.6. Quantitative Real-Time PCR Analysis (QRT-PCR)

One-microgram total RNA was used for the first-strand cDNA synthesis, according to the manual of RevertAid™ First Strand cDNA Synthesis Kit (Thermo Scientific, Waltham, MA, USA). The primers for QRT-PCR analysis were designed by using AlleleID 6.0 (https://www.softpedia.com/get/Science-CAD/AlleleID.shtml, accessed on 5 August 2019). β-actin was used as the reference gene. Details about the primers for QRT-PCR were listed in Table S1. The QRT-PCR in a total volume of 20 μL included 0.2 μM primers, 50 ng cDNA, and 10 μL 2 X SYBR Green Fast qPCR Mix (Biomarker, Beijing, China). The QRT-PCR was performed on the Bio-Rad CFX96 system (BioRad lab Inc., Hercules, CA, USA), with a protocol of 95 °C for 3 min, 95 °C for 5 s and 60 °C for 30 s for 45 cycles. The relative expression for QRT-PCR analysis was calculated by using 2^−ΔΔCt^ method [18]. 

### 2.7. Metabolites Extraction and Detection

The extraction of metabolites was performed according to the study by Ma et al. [19], with a little modification. In total, 100 mg freeze-dried leaf powder was extracted with 70% aqueous methanol at 4 °C overnight, then centrifuged at 10,000× *g* for 10 min. The extracts were absorbed with 3 mL Carbon-GCB SPE Cartridge and filtrated with 0.22 μm membrane. The 2-chlorophenylalanine dissolved in dimethyl sulfoxide was used as the internal standard. 

The extracts were detected and identified by the Metware biotech company (Wuhan, China). A total of 4 μL extract was injected in an ultra-performance liquid chromatograph (UPLC, Shimadzu, Japan) carrying a Waters ACQUITY UPLC HSS T3 C18 column (1.8 µm × 2.1 mm × 100 mm). The column temperature was 40 °C. Ultrapure water and acetonitrile, including 0.04% acetic acid, were used as mobile phase A and B, respectively. The gradient elution procedure was conducted as follows: 5% B at 0 min, up to 95% in 9 min, maintaining for 1 min, then decreased to 5% in 1 min, and maintaining for 3 min. The total flow rate was 0.35 mL/min. After electrospray ionization at 550 °C, 5500 V and 30 psi, the metabolites were identified by Applied Biosystems 4500 QTRAP tandem mass spectrometry using MRM scan mode. The qualitative analysis of metabolites was conducted based on the MWDB database (Metware biotech) and public database. In order to improve accuracy of qualitative and quantitative analysis, the mass spectrum peaks of some metabolites were selected randomly and re-calibrated according to retention indexes and mass spectra. In addition, the mixture of all the sample extracts was injected per 10 samples to evaluate the stability of the HPLC-MS analysis. Quantification of the identified metabolites was conducted by using Analyst 1.6.3. Variable Importance in Projection ≥ 1 and 0.5 ≥ Fold change ≥ 2 were used as the threshold for selecting differential metabolites. 

### 2.8. Statistical Analysis

The statistical analysis was performed using Excel 2016 and Graphpad Prism 5.0. One-way ANOVA was used for pairwise comparisons; *p* < 0.05 was considered to be significantly different.

## 3. Results

### 3.1. Flavor Components of Changqing Tea

**Figure 1:**
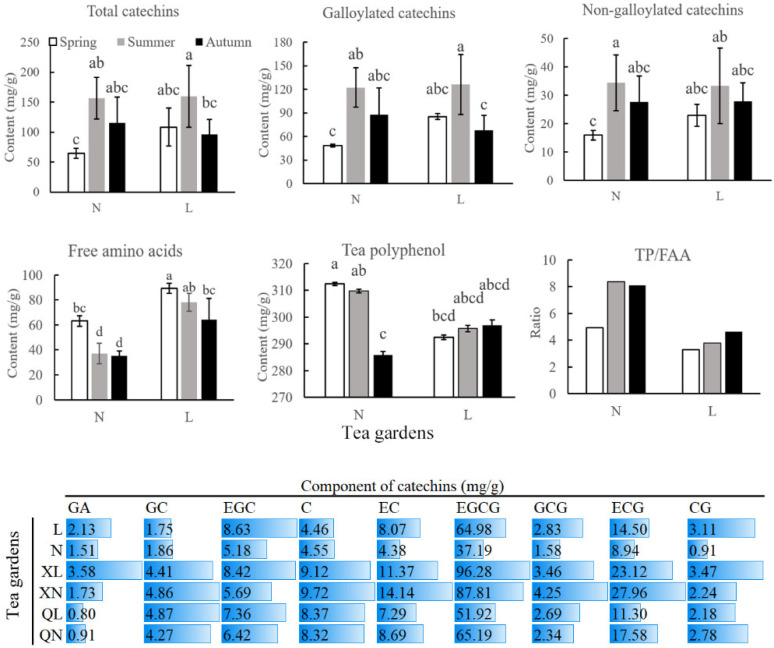
Flavor components of Changqing tea in different seasons. Different lowercase letters represent different significantly on *p* < 0.05 level. N, XN, and QN mean samples from Nanhuyulu in spring, summer and autumn, respectively. L, XL, and QL mean samples from Litaishan in spring, summer and autumn, respectively. GA, gallic acid; GC, gallocatechin; EGC, epigallocatechin; C, catechin; EC, epicatechin; EGCG, epigallocatechin gallate; GCG, gallocatechin gallate; ECG, epicatechin gallate; CG, catechin gallate; TP, tea polyphenol; FAA, free amino acids.

In Changqing tea, the total catechins was 65–160 mg/g, with 16–34 mg/g non-galloyated catechins and 49–126 mg/g galloylated catechins. Tea polyphenols and free amino acids account for 286–312 mg/g and 35–89 mg/g, respectively. Of the catechins, epigallocatechin gallate (EGCG) was the highest, with mean content of 37–96 mg/g. Tea polyphenols, total catechins, galloylated catechins and non-galloylated catechins showed higher contents in summer, compared to that in spring. Meanwhile, these components decreased in autumn, but most of them were still higher than that in spring. Free amino acids revealed reverse trend with catechins and polyphenol; it showed the highest level in spring and decreased in summer and autumn. (Figure 1).

### 3.2. Identification of Differential Expressed Genes 

At least 5.82 Gb clean bases were obtained for each sample with GC content of 45.29–45.72% and Q30 of 93.91–95.29% (Table 1). The clean data have been submitted to the SRA database with accession of PRJNA830980. More than 84.73% sequences could be mapped onto the tea genome, and more than 72.40% unique mapped reads were obtained, which revealed the high quality of these sequencing data.

**Figure 2:**
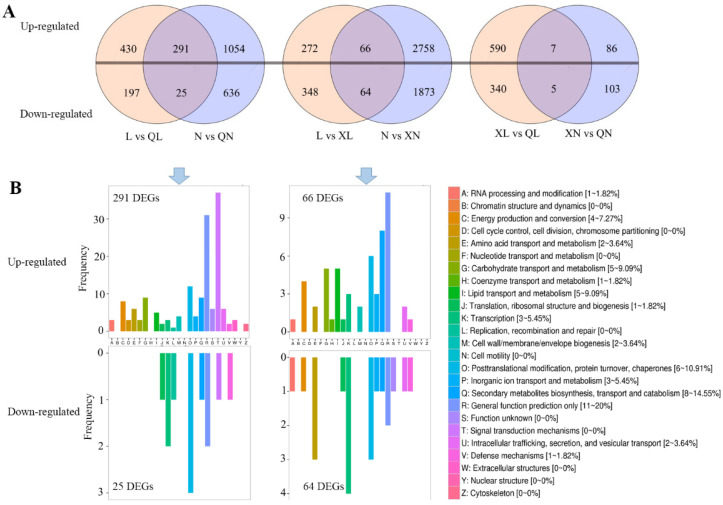
Differential expressed genes of Changqing tea leaves during different seasons. (**A**), the Venn diagram of differential expressed genes during different seasons. (**B**), the KOG classification of common differential expressed genes in the samples from two tea gardens between different seasons. N, XN, and QN mean samples from Nanhuyulu tea garden in spring, summer, and autumn, respectively. L, XL and QL mean samples from Litaishan tea garden in spring, summer, and autumn, respectively.

In order to elucidate the molecular mechanism of Changqing tea flavor, the transcriptome of the tender leaves under different seasons was sequenced. The results revealed 316, 130, and 12 DEGs in comparisons of spring vs. autumn, spring vs. summer, and summer vs. autumn, respectively (Figure 2A). Based on DEG analysis, the difference in transcript expression of spring vs. autumn and spring vs. summer was larger than summer vs. autumn. KOG annotation classification revealed that most of DEGs for spring vs. autumn were enriched in “signal transduction mechanism”, “general function prediction only”, and “posttranslational modification, protein turnover, chaperones”. For comparison of spring and summer, “general function prediction only”, “secondary metabolites biosynthesis, transport and catabolism”, and “posttranslational modification, protein turnover, chaperones” enriched the most DEGs (Figure 2B).

QRT-PCR analysis revealed an expected result that the relative expression patterns of DEGs during different seasons were consistent with the RNA-Seq data (Figure S1).

### 3.3. Identification of Differential Metabolites

**Figure 3:**
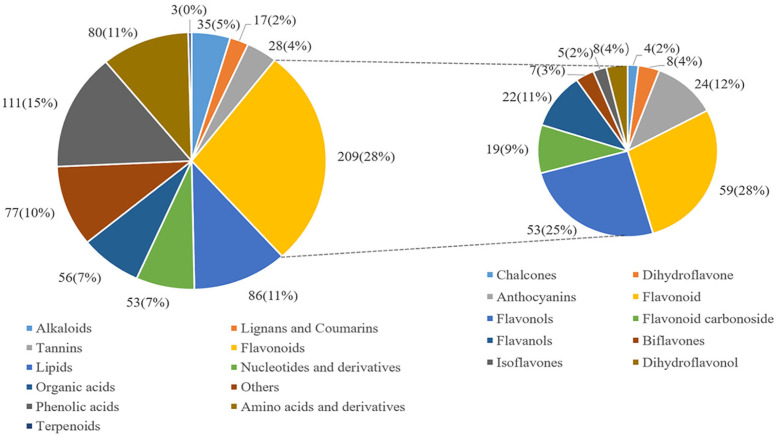
Classification of metabolites identified in Changqing tea shoots.

A total of 755 metabolites from tea leaves of Changqing tea were identified, including 209 flavonoids (28%), 111 phenolic acids (15%), 86 lipids (11%), 80 amino acids and derivatives (11%), etc. (Figure 3). Among flavonoids, flavonoid, flavonols, and flavanols account for 28%, 25%, and 11%, respectively.

**Figure 4:**
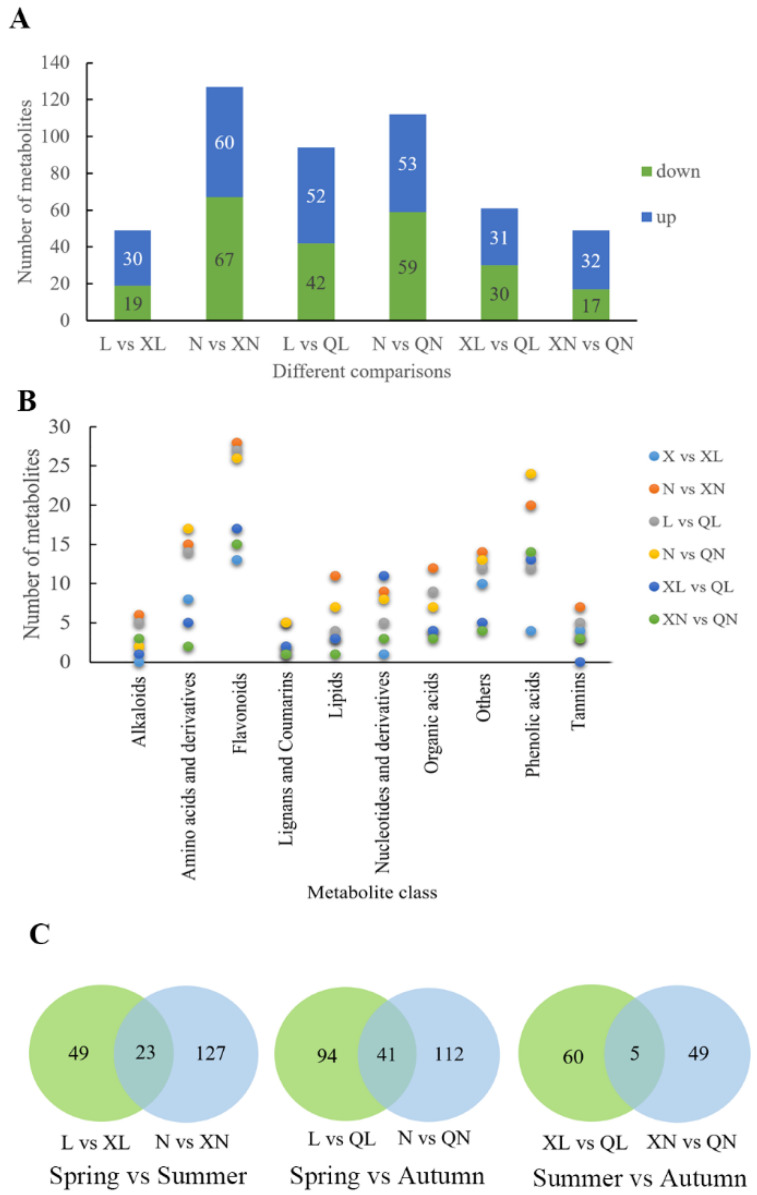
Differential metabolites identified in Changqing tea shoots during different seasons. (**A**), Differential metabolites numbers of Changqing tea shoots in different seasons from two tea gardens. (**B**), Classification of differential metabolites in Changqing tea shoots during different seasons. (**C**), Venn diagram of differential metabolites in Changqing tea shoots during different seasons. N, XN and QN mean samples from Nanhuyulu tea garden in spring, summer, and autumn, respectively. L, XL and QL mean samples from Litaishan tea garden in spring, summer, and autumn, respectively.

For different season comparisons, 49, 127, 94, 112, 61, and 49 differential metabolites were identified in L vs. XL, N vs. XN, L vs. QL, N vs. QN, XL vs. QL, and XN vs. QN, respectively (Figure 4A and Table S2). For differential metabolites, flavonoids, phenolic acids, and amino acids and derivatives accounted for the largest number, which were the most important constituents of tea flavor (Figure 4B). Furthermore, the difference of tea metabolome between summer and autumn was smaller than other season comparisons. A Venn diagram showed the similar result that summer vs. autumn comparison identified 5 differential metabolites, which was much less than 23 differential metabolites in spring vs. summer and 41 differential metabolites in spring vs. autumn (Figure 4C and Table S3).

### 3.4. Flavonoid Biosynthesis of Changqing Tea in Different Seasons

In comparison of spring and summer, two flavonoid 3′-hydroxylase genes (F3′H, CSS0032858, CSS0014209) were found to be up-regulated in summer. Flavonoid 3′-hydroxylase belong to the cytochrome P450 super family, catalyze flavonoid hydroxylation at the 3′ positions of the B-ring and promote biosynthesis of cyanidin- anthocyanins, flavonols (quercetin and myricetin), and flavanols (catechin and epicatechin). Compared to spring, catechin and epicatechin of Changqing tea were increased in summer (Figure 1). As well, Quercetin-3-O-(2″-Galloyl-Alpha-L-Arabinoside) was also increased in summer compared to spring (Table S3), which showed a similar trend with F3′H genes expression.

In autumn, epicatechin showed similar content to spring, but catechin was still higher than spring (Figure 1). Meanwhile, Quercetin-3-O-(2″-Galloyl-Alpha-L-Arabinoside) in autumn was also higher than in spring. However, the expression of F3′H genes was not different significantly in comparison of spring and autumn. In addition, lignin-biosynthesis-related genes cinnamyl-alcohol dehydrogenase (CAD, CSS0049172) and peroxidase (POD, CSS0011466, CSS0028286, CSS0050018) were up-regulated in autumn.

Comparing summer to autumn, for flavonoids, only Cyanidin-3-p-coumaroylrutinoside-5-glucoside was decreased in autumn. Moreover, no significant changes were found in flavonoid biosynthesis-related genes (Table S3).

### 3.5. Amino Acid Metabolism of Changqing Tea in Different Seasons

In comparison of spring and summer, 6 differential amino acids and derivatives were identified, including L-Asp (−1.16), L-threo-3-Methylaspartate (−1.18), L-His (−2.11), 4-Hydroxy-L-Glu (−1.05), H-HomoArg-OH (−3.62), and S-(Methyl)glutathione (−1.49), all of which were decreased in summer. Most of these amino acids impart umami taste. In addition, three genes contributing degradation of Leucine, Valine, and Isoleucine were up-regulated in summer, including branched-chain amino acid aminotransferase (BCAT, CSS0023496), 2-oxoisovalerate dehydrogenase (BCKDHA, CSS0022049), and dihydrolipoamide branched chain transacylase (DBT, CSS0044303). The presence of Val and Leu in peptides imparted bitterness of tea. Although no significant changes were found in Val and Leu content from Changqing tea in different seasons, the three amino acid metabolism related genes played potential roles in regulating tea flavor. It is worth noting that S-(Methyl)glutathione, which is a kokumi active compound, was also decreased in summer.

**Figure 5:**
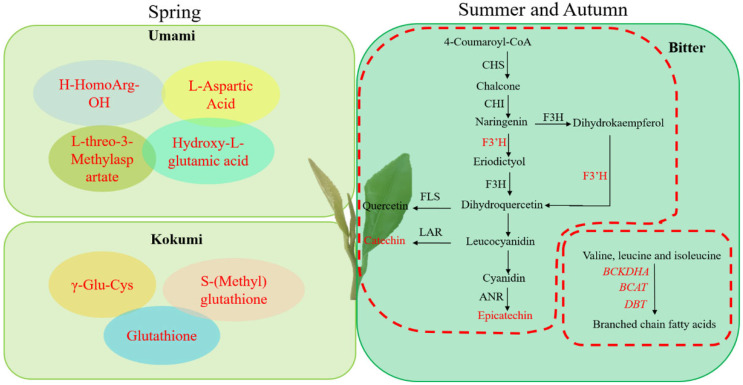
The potential mechanism of flavor changes of Changqing tea during different seasons. Red letters represent up-regulated DEGs or metabolites.

In autumn, 8 amino acids were decreased compared to spring, including L-Histidine (−2.58), L-Homomethionine (−1.17), H-HomoArg-OH (−3.81), 2,6-Diaminooimelic acid (−2.06), γ-Glu-Cys (−1.97), reduced Glutathione (−2.66), S-(Methyl)glutathione (−1.88), and S-(5′-Adenosy)-L-homocysteine (−1.13). Except for S-(Methyl)glutathione, Glutathione and γ-Glu-Cys also belong to kokumi taste-contributing amino acids. It means that the kokumi and umami taste of Changqing tea in spring is stronger than in summer and autumn (Figure 5).

## 4. Discussion

In this study, in order to clarify the molecular mechanism of the flavor of Changqing tea, transcriptome and metabolome of tender shoots from two tea garden in Changqing were conducted. The results showed that Changqing tea contained high free amino acids and tea polyphenols. In addition, catechins and tea polyphenols of Changqing tea were less in spring than in summer and autumn, but free amino acids were higher in spring. Correspondingly, the flavonoid and lignin biosynthesis related genes showed higher expression levels in summer and autumn. Meanwhile, compared to spring, amino acids with bitter taste were higher in summer and autumn. The flavonoid changes of Changqing tea in different seasons were consistent with the study by JIANG et al. [20].

Tea polyphenols are one of the most important flavor components, which possesses antioxidant activity [21,22], antimicrobial properties [23,24], and anti-cancer activity [25,26]. In tea shoots, catechins were the major constituents of tea polyphenols. In general, tea plants with albino leaves contained lower tea polyphenols or catechins than green tea cultivars. It leads to the slight bitter taste of albino tea leaves. For examples, green tea cultivars Fudingdabai and Zhenong 113 possessed total catechins of 150 and 16.8 mg/g, respectively. However, the albino tea cultivars Xiaoxueya, Baiye 1, and Huangjinya possessed total catechins of 132 mg/g, 100–123 mg/g, and 130 mg/g, respectively [27,28]. In spring, Changqing tea showed total catechins of 65–111 mg/g, which was at the level of catechins in albino tea cultivars. In summer and autumn, the total catechins of Changqing tea was at the level of normal green tea cultivars. Nevertheless, Changqing tea showed high content of tea polyphenols, with 286–312 mg/g during all the three seasons, which was significantly higher than albino tea cultivar Baiye 1, with 114–262 mg/g in different developmental stages [29], and purple tea cultivars with 188–246 mg/g for the unaerated tea [30]. This result indicated that the antioxidant activity of Changqing tea was not reduced due to the low content of catechins.

Transcriptome analysis revealed that F3′H was up-regulated in summer compared with spring, then resulted in accumulation of catechin and epicatechin in summer. Therefore, Changqing tea showed more bitter taste in summer than in spring. In autumn, the expression of F3′H had no difference compared to spring, but lignin biosynthesis related genes such as CAD and POD were up-regulated. This result indicated more lignins were synthesized in the tea shoots in autumn, suggesting a more tender taste of Changqing tea in spring.

Amino acids are another major component which determines the quality of tea. Its composition and content were affected by environmental factors, such as light, temperature, and soil fertilizers, etc. [7,31]. In Changqing tea, the free amino acids in spring was 63–89 mg/g, which was higher than albino tea cultivars Xiaoxueya of 41 mg/g and Baiye 1 of 35 mg/g, Huangshanbaicha of 71 mg/g during the albinostic stage, as well as higher than green cultivars of Zhenong 113 of 30 mg/g [27,32]. The high amino acids give the tea an umami taste. Different amino acids possessed different tastes: Pro, Gly, Ala, Val, Leu, Tyr, and Phe imparted the bitter taste; Glu, Asp, Tyr, pyro-Glu and succinyl amino acids (such as suc-Arg and suc-Glu) imparted an umami taste; glutathione and γ-Glutamyl dipeptides were kokumi active compounds [33]. In this study, the umami-taste -related amino acids were decreased in summer compared to spring, such as Asp and Glu. Meanwhile, three genes involved in metabolism of Leu, Val, and Iso-Val, which were bitter-taste-contributing amino acids, were up-regulated in summer. These results demonstrated that the taste of Changqing tea was bitter in summer and autumn than in spring. Nevertheless, the total free amino acids of Changqing tea in summer and autumn was similar to albino tea cultivars in spring. This might be a major reason for the slight bitter taste of Changqing tea.

To our knowledge, this is the first report about the kokumi taste in tea. Metabolome analysis showed that some kokumi taste active compounds were conspicuously accumulated in spring in Changqing tea, such as glutathione, γ-Glu-Cys, and S-glutathione. Except for the role of kokumi taste active compounds, high total polyphenols and high free amino acids emphasized the thickness of the tea flavor, and then resulted in the kokumi taste of Changqing tea.

It had to be explained that the great flavor of Changqing tea was largely caused by the climate of its plant area. In Changqing District, the existing tea cultivars were introduced from south of China. In the present study, the Fudingdabai tea cultivar was used, which is not only the most widely planted tea cultivar in Changqing District, but also widely planted throughout China. After a long period of domestication, it exhibited considerable quality changes from the original region. Above all, the high-latitude and big temperature difference between day and night in Changqing District moulds the flavor of Changqing tea.

## 5. Conclusions

In conclusion, Changqing tea contains an enriched high content of polyphenols and high free amino acids. The lower expression of flavonoid biosynthesis related genes produced less catechins, and the higher accumulation of umami and kokumi amino acids constructed the special flavor of Changqing tea.

## Figures and Tables

**Table 1:** Quality and genome alignment efficiency of sequencing data.

Sample ID	Total Reads	GC Content	Q30	Mapped Reads	Uniq Mapped Reads
L1	44,484,588	45.35%	94.84%	38,627,832 (86.83%)	32,337,944 (72.69%)
L2	46,058,340	45.37%	94.89%	40,035,790 (86.92%)	33,536,472 (72.81%)
L3	38,975,414	45.72%	94.49%	33,881,495 (86.93%)	28,279,355 (72.56%)
N1	44,248,808	45.38%	94.45%	38,350,376 (86.67%)	32,204,533 (72.78%)
N2	42,729,226	45.52%	94.51%	36,935,621 (86.44%)	30,977,168 (72.50%)
N3	39,548,916	45.51%	94.20%	34,339,197 (86.83%)	28,631,767 (72.40%)
XL1	43,648,802	45.51%	94.78%	37,879,990 (86.78%)	31,889,023 (73.06%)
XL2	43,559,986	45.67%	94.84%	37,926,766 (87.07%)	32,015,677 (73.50%)
XL3	43,038,378	45.49%	94.82%	37,442,835 (87.00%)	31,632,998 (73.50%)
XN1	43,957,652	45.58%	94.51%	38,161,588 (86.81%)	32,217,373 (73.29%)
XN2	41,767,546	45.29%	93.91%	36,196,630 (86.66%)	30,599,340 (73.26%)
XN3	43,149,206	45.43%	94.94%	37,390,956 (86.66%)	31,694,135 (73.45%)
QL1	48,320,578	45.43%	95.29%	41,721,506 (86.34%)	35,084,628 (72.61%)
QL2	53,669,856	45.58%	94.97%	46,314,442 (86.30%)	38,931,575 (72.54%)
QL3	52,343,966	45.51%	94.32%	44,353,491 (84.73%)	37,160,579 (70.99%)
QN1	41,956,338	45.65%	94.44%	36,024,372 (85.86%)	30,405,207 (72.47%)
QN2	44,766,142	45.29%	94.29%	38,612,001 (86.25%)	32,659,135 (72.95%)
QN3	42,372,422	45.49%	94.12%	36,598,453 (86.37%)	30,941,615 (73.02%)

## Data Availability

The transcriptomic data has been deposited in SRA database with accession of PRJNA830980.

## Supplementary Materials

The following supporting information can be downloaded at: https://www.mdpi.com/article/10.3390/foods11152289/s1, Table S1: The primer used for QRT-PCR analysis; Table S2: The differential metabolites of Changqing tea in different seasons; Table S3: The common differential metabolites of Changqing tea in both tea gardens between different seasons; Figure S1: QRT-PCR verification of differential expressed genes.
