# Supplementary material for: Molecular Link in Flavonoid and Amino Acid Biosynthesis Contributes to the Flavor of Changqing Tea in Different Seasons
Source: Foods. 2022 Jul 31;11(15):2289. doi: 10.3390/foods11152289 (PMC9368528; doi:10.3390/foods11152289)
Supplement: Supplementary file 1 [file foods-11-02289-s001.zip › Table S1.pdf]

Table S1. The primer used for QRT-PCR analysis.

Gene ID	Primer pairs	Tm	GC%	Product (bp)
CSS0016564	AGCACATTCTCAAGACCAACTTC	56.9	43.5	140
	ACTCGTGACTCGCTAACTTCC	57.1	52.4	
CSS0005847	CTAACCCATCCAACAACCAACC	56.9	50	110
	CCGCAGAGATTGAGAGGAAGG	57.4	57.1	
CSS0013230	AATACTTCACCGATGAGTTCTGG	55.9	43.5	130
	CACCTTGTAATTCCCTTCTCTCC	56.2	47.8	
CSS0039075	TCTCCACCGTTGCCATATCC	56.5	55	155
	GCGATGTGTTGTTGTTGTTGG	56.5	47.6	
CSS0029164	TTGTCTTGACCGCCTCTGATG	57.6	52.4	136
	GCACTGAGTCTCCGAGTTACC	57.4	57.1	
CsGAPDH	TGGGTGTCAATGAGAAGGATTAC	60.2	43.5	
	TTTGTGTGGCTGTGATGGAG	60.7	50	114
