# Supplementary figures and images for: Molecular Link in Flavonoid and Amino Acid Biosynthesis Contributes to the Flavor of Changqing Tea in Different Seasons
Source: Foods. 2022 Jul 31;11(15):2289. doi: 10.3390/foods11152289 (PMC9368528; doi:10.3390/foods11152289)

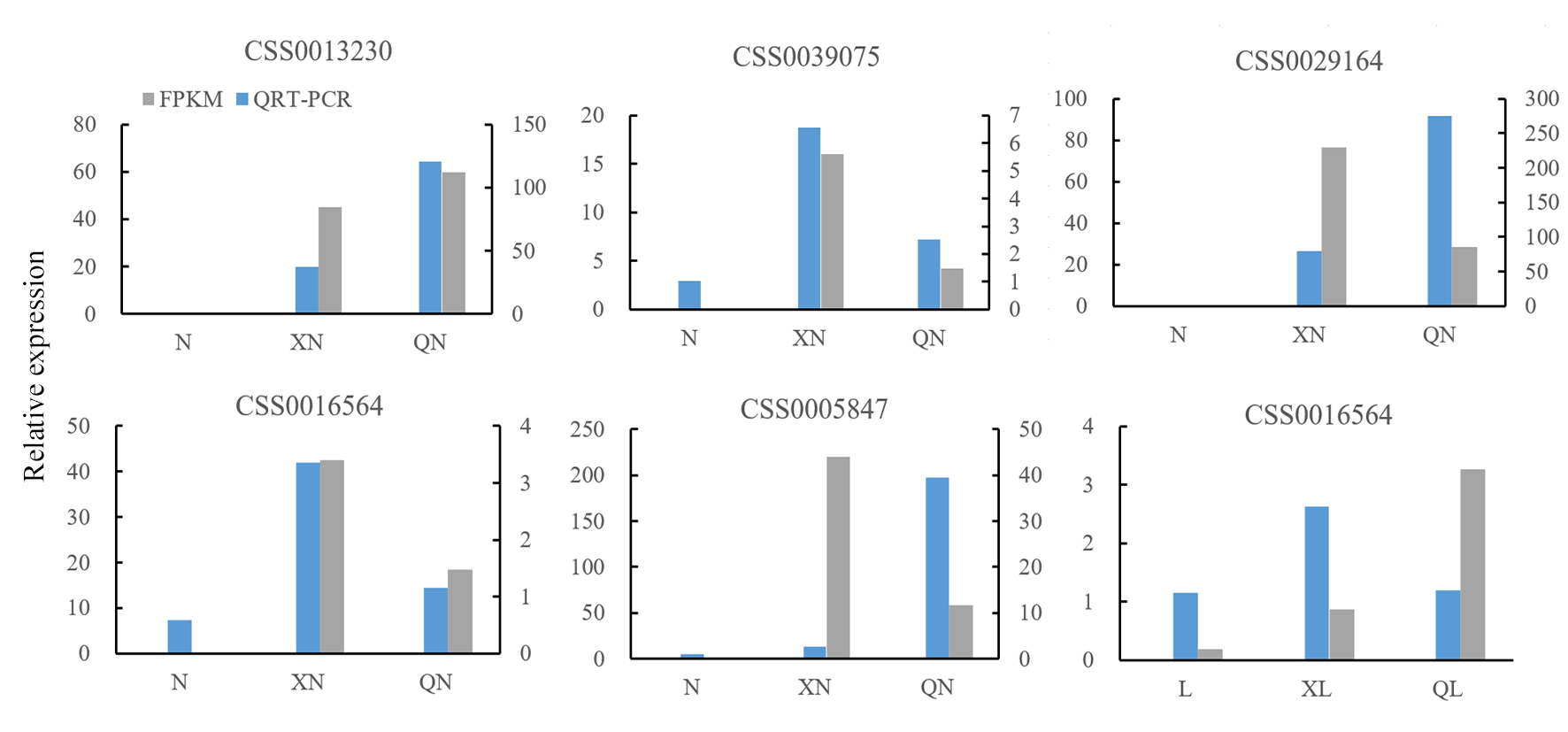

Supplement: Supplementary file 1 [file foods-11-02289-s001.zip › Figure S1.tif]
